# Postoperative Complications of Impacted Mandibular Third Molar Extraction Related to Patient's Age and Surgical Difficulty Level: A Cross-Sectional Retrospective Study

**DOI:** 10.1155/2022/7239339

**Published:** 2022-01-03

**Authors:** Andra Rizqiawan, Yeni Dian Lesmaya, Anindita Zahratur Rasyida, Muhammad Subhan Amir, Shigehiro Ono, David Buntoro Kamadjaja

**Affiliations:** ^1^Department of Oral and Maxillofacial Surgery, Faculty of Dental Medicine, Universitas Airlangga, Surabaya, Indonesia; ^2^Department of Oral and Maxillofacial Surgery, Graduate School and Institute of Biomedical and Health Sciences, Hiroshima University, Hiroshima, Japan

## Abstract

Mandibular third molar surgical extraction, either partially erupted or fully impacted, is the most common surgical procedure in oral and maxillofacial surgery (OMFS). However, this procedure can be associated with many postoperative complications including persistent pain, swelling, trismus, and paresthesia due to nerve injury. This study aimed to identify the correlation of postoperative complications with patient's age, sex, and surgical difficulty level. This study was a cross-sectional retrospective and single-center research conducted on patients with a history of mandibular third molar surgical extraction in the period between 2017 and 2019 at Dental and Oral Hospital Universitas Airlangga, Surabaya, Indonesia. The researchers assessed the factors of age, sex, and surgical difficulty level regarding postoperative complications on the first day of the surgery and after one week on the 7th day of it. Among 916 respondents, the majority of the sample was females (59%) and the dominant age group (60.9%) was the age group of 21–30 years while the dominant surgical difficulty level was shown by the advanced cases group (77%). The statistical analysis showed that there was a significant correlation between surgical difficulty level and postoperative complications including pain, trismus, and paresthesia on the first-day assessment. On the other hand, age was significantly related to complications like pain, swelling, and trismus on the first-week assessment. Age and surgical difficulty level were the most common risk factors of the mandibular third molar extraction postoperative complications. Dentists should take into consideration that older patients (≥51 years) and patients with complex surgical level are more vulnerable to severe postoperative complications.

## 1. Introduction

Tooth impaction is the condition in which a tooth fails to fully erupt from the gums within the expected time [1]. The surgical procedure of removing the impacted tooth is known as odontectomy, and it is very common among mandibular third molars since the highest percentage of the impacted teeth is seen among mandibular third molars (M3) [2]. However, odontectomy is considered the most common procedure in whole oral and maxillofacial surgery (OMFS) [3].

Postoperative complications rate of impacted mandibular third molar extraction varies between 2.6% and 30.9% including bleeding, swelling (edema), persistent pain, trismus, and nerve injury. Recent studies have considered patient characteristics such as age and sex, impacted tooth level, surgical techniques, and operator skills as risk factors of postoperative complications [4]. Therefore, it is crucial to analyze the surgical difficulty level before performing the procedure to estimate its success rate.

Based on the abovementioned risk factors, the researchers have observed the demographic data of patients with mandibular third molar surgery history associated with high-frequency visits at the dental and oral university hospital. The study observed the demographic data including patients' age and sex and analyzed the mandibular third molar surgical difficulty level. The observation and analysis results were then used to determine the correlation of these variables with the risk of postoperative complications.

## 2. Materials and Methods

**Figure 1:**
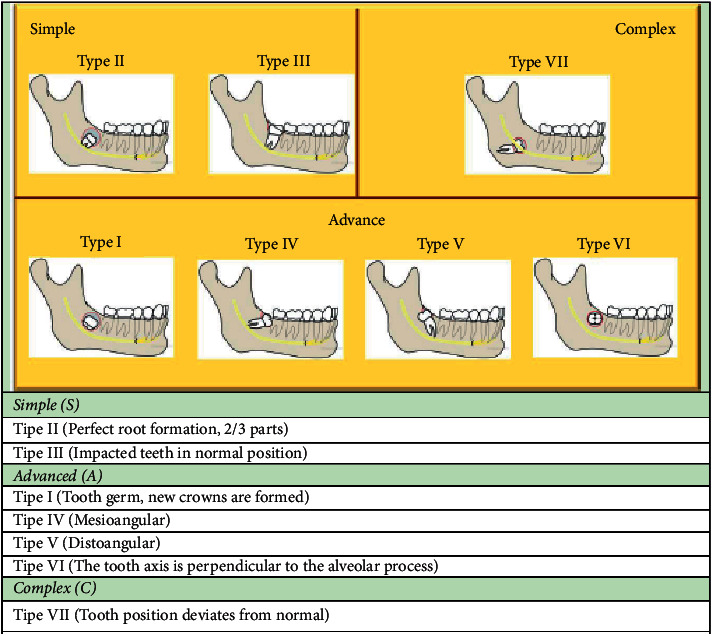
Classification of surgical difficulty level of mandibular third molar extraction according to the type of retention based on Sailer and Pajarola [6].

This study was a two-year cross-sectional retrospective (2019-2020) and single-center research study at Dental and Oral Hospital of Universitas Airlangga (RSGM Unair), Surabaya, Indonesia. Ethical approval was obtained from the Ethics Committee of the Faculty of Dental Medicine, Universitas Airlangga, with number 679/HRECC.FODM/X/2019. The research instruments were patients' medical records in addition to patients' panoramic photos needed to support the diagnosis, collected by 2 researchers who have been trained through pilot study with a small-scale sample number. The research sample included patients with a history of impacted mandibular third molar surgical extraction using local anesthesia and split technique using a low-speed straight handpiece in the period between July 2017 and July 2019. On the other hand, patients with incomplete medical records (referring to patients who failed to follow the postoperative evaluation according to the schedule), without impacted tooth X-rays, and with the absence of craniofacial anomalies, congenital anomalies, and accompanying syndromes were excluded from the sample. Based on the first-day preoperative assessment and the seventh-day postoperative assessment, the researchers cross-sectionally analyzed the patients' data to point out the postoperative complications such as persistent pain, swelling, trismus, and paresthesia in relation to patients' characteristics such as age and sex and surgical difficulty level. Postoperative pain rates were measured according to the visual analog scale (VAS). Edema was measured by comparing the difference of preoperative measurement and postoperative measurement using a ruler according to Soylu et al. [5]. Trismus was evaluated by measuring maximum interincisal opening. The surgical difficulty level of the impacted mandibular third molar extraction was classified based on the type of retention according to Sailer and Pajarola [6] (Figure 1). The descriptive data were analyzed using the Chi-Square test with a significant value *P* < 0.05 with IBM Statistical Package for the Social Sciences (SPSS), Version 25.0 (IBM Corp., Armonk, New York, USA).

## 3. Results

The total sample included 916 patient medical records. The data showed that 59% of the respondents were females (*n* = 540), while the dominant age range was 21–30 years with a percentage of 60.9% (*n* = 558). Moreover, surgeries for impacted left mandibular third molars were slightly more than those of the right molars, with a percentage of 50.5% (*n* = 463). Based on the type of retention [6] (Figure 1), 20.9% were simple cases (*n* = 191), 77% were advanced cases (*n* = 705), and 2.2% were complex cases (*n* = 20). The data distribution based on sex is listed in Table 1.

Data showing postoperative complications of the impacted mandibular third molars based on age groups are listed in Table 2. Although the data showed no significant difference among all age groups, the most common postoperative complication on the first-day assessment was the pain for the age group 21–30 years (*P* ≥ 0.001) with a percentage of 87% in the whole age group. Although the pain complication value has declined from first-day assessment to the first-week assessment, the latter showed significant results in the persistent pain complaint (*P* ≥ 0.001) in the age groups of 11–20 years, 21–30 years, and 51–60 years with percentages of 1.8%, 1.3%, and 12.5%, respectively. Moreover, the first-day assessment showed no significant difference in swelling complications (edema) in all age groups, while the first-week assessment presented that the percentage of complications in the age group of 11–20 years was the highest (2.9%) compared to other age groups. Furthermore, trismus complications were primarily reported in all age groups on the first-week assessment. On the other hand, paresthesia complications were not correlated with all age groups.

Based on sex data, there was no significant difference between male and female postoperative complications such as pain, swelling, trismus, and paresthesia whether on first-day or first-week assessment (Table 3). However, according to surgical difficulty level, the first-day assessment showed that the advanced level group complained mostly from postoperative pain (91.3%) while the complex level group suffered mainly from trismus (55%) and the simple level group experienced paresthesia for the most part (20%) (Table 4).

## 4. Discussion

Recent studies have shown that swelling, pain, and trismus are possible transient complications following the odontectomy [4] due to the physiological inflammation caused by tissue response to surgical manipulation and trauma. Even though they are considered normal and treatable conditions, dentists should be aware of any abnormality which may lead to postoperative infection or prolonged complications [7, 8].

Postoperative pain begins once the local anesthetic effect has worn off and reaches the peak within 6 to 12 hours after the surgery. This is similar to this study's first-day assessment which demonstrated that all age groups from both sexes complained of postoperative pain. Moreover, unpredictably, the first-day assessment showed that the complex surgical difficulty group had the most pain complaint (80%) compared to all groups despite taking the same analgesics. On the other hand, the first-week assessment presented that postoperative pain has relieved significantly in all age groups except in the age group of 51–60 years. Moreover, swelling complications showed a remarkable drop from the first-day assessment to the first-week assessment. However, the age group of 51–60 years reported having swelling at day 7, followed by persistent postoperative complications including pain and trismus due to the slow inflammatory response in the older age group compared to other younger age groups. Similarly, the existing theories state that the inflammatory response cells, macrophages, T cells, and mesenchymal stem cells are exposed to intrinsic age-related changes that could impact, along with vascularization and angiogenesis impairment, the elderly healing process [6, 9]. Furthermore, old age is accompanied by the activity of osteochondral cells and their progenitors in addition to weaker defense against foreign organisms, thereby being more susceptible to infections due to the lymphoid tissue changes including T cells that eventually will influence the antibodies' production [10, 11]. Finally, compared to young patients, old patients have denser bone and more complete root development in addition to a higher probability of ankylosing spondylitis as to why old patients need a longer duration of bone removal during the surgery [12].

Based on the surgical difficulty level, the first-day assessment showed that trismus was associated with the complex cases group. Major factors in terms of a deeper impacted tooth, a higher number of overlaying bones, and a greater angulation can increase the extraction difficulty which eventually causes more trauma [7]. However, masticatory myositis may appear as a secondary complication of this trauma [13]. Since high surgical difficulty level can be associated with a masticatory musculature-prolonged extreme stretching during the surgery. There is a strong correlation between postoperative pain and trismus, indicating that pain may be one of the principal reasons for the limitation of opening after the removal of impacted third molars [4].

Besides, the first-day assessment showed that paresthesia was associated with the complex cases group. This is because of factors such as extensive trauma and the tooth's depth and distance to the alveolar canal that may increase the inferior alveolar nerve trauma risk, thereby causing paresthesia [4, 14]. Although studies showed that sex did not influence the nerve healing process, others approved that age can do. Nerve damage can be permanent, or for more than 6 months, and it varies among mild hypoesthesia, complete anesthesia, or neuropathic response which causes chronic pain [15]. Treatment of this complication can be approached through nonsurgical methods, such as Vit B complex, corticosteroid, laser therapy, and acupuncture and surgery. Several reports suggested a higher chance of spontaneous reinnervation and recovery of the nerve within the inferior alveolar canal [4].

Several studies stated sex as a determinant of postoperative complications risk. Some of them showed that males had a lower pain risk than females [16, 17]. However, this research presented no significant difference in postoperative complications among males and females.

In the end, this research had some limitations in terms of the used data. The duration of surgery time was not evaluated in this study which may be a crucial variable in complication after third molar surgery. Aside from that, the surgery was done by more than 1 surgeon who may vary in skill and technique that might interfere the overall outcome. Different kinds of flaps, suture techniques, and tightness may contribute to swelling after surgery, whereas tight closure increases postoperative swelling and pain for difficult draining [4]. When the extraction technique is getting harder, surgery duration is getting longer and this condition leads to more edema and trismus [5]. In future studies, it is recommended to include more variables such as the correlation of the impacted mandibular third molar with the infra-alveolar nerve using radiographic examination, root number, and root morphology, social history elements such as smoking, oral contraceptive medicine intake, intraoperative complications, surgical techniques, and surgeon's experience, as well as the operation duration.

## 5. Conclusion

According to the research results, there was a significant correlation between mandibular third molar surgical extraction and postoperative complications including pain, swelling, trismus, and paresthesia concerning patients' age and surgical difficulty level. It has been concluded that the older age group of ≥51 years and complex cases group had the highest complication risk.

## Figures and Tables

**Table 1:** Data distribution based on sex.

Variable	Total	(%)
Gender		
Female	540	59.0
Male	376	41.0
Age		
11–20	170	18.6
21–30	558	60.9
31–40	137	15.0
41–50	41	4.5
51–60	8	9
61–70	2	2
Impacted tooth		
GIGI 38	463	50.5
GIGI 48	453	49.5
Impaction type		
Half	781	85.3
Total	135	14.7
Level position		
I A	94	10.3
II A	587	64.1
III A	15	1.6
I B	5	5.0
II B	187	20.4
III B	15	1.6
I C	0	0
II C	11	1.2
III C	2	0.2
Angulation		
Vertical	195	21.3
Mesioangular	386	42.1
Distoangular	43	4.7
Horizontal	286	31.2
Buccoversion	6	0.7
Distoversion	0	0
Inverted	0	0
Retention type		
Simple	191	20.9
Advanced	705	77.0
Complex	20	2.2

**Table 2:** Comparison of mandibular third molar extraction postoperative complications based on age.

Age (*N*)	Complications							
	Pain		Edema		Mouth opening restriction		Paresthesia	
	Day-1	Day-7	Day-1	Day-7	Day-1	Day-7	Day-1	Day-7
11–20 (170)	152 (89.4%)	3 (1.8%)	146 (85.9%)	5 (2.9%)	48 (28.2%)	3 (1.8%)	1 (0.6%)	1 (0.6%)
21–30 (558)	490 (87.8%)	7 (1.3%)	453 (81.2%)	8 (1.4%)	148 (26.5%)	3 (0.5%)	14 (2.5%)	4 (0.7%)
31–40 (137)	120 (87.6%)	0 (0%)	106 (77.4%)	0 (0%)	37 (27%)	1 (0.7%)	1 (0.7%)	0 (0%)
41–50 (41)	38 (92.7%)	0 (0%)	31 (75.6%)	0 (0%)	13 (31.7%)	0 (0%)	0 (0%)	0 (0%)
51–60 (8)	7 (87.5%)	1 (12.5%)	6 (75.0%)	2 (25%)	3 (37.5%)	1 (12.5%)	1 (12.5%)	0 (0%)
61–70 (2)	2 (100%)	0 (0%)	1 (50%)	0 (0%)	0 (0%)	0 (0%)	0 (0%)	0 (0%)
*P* values	≥0.01	≥0.01	≥0.01	≤0.001	≥0.01	≥0.01	≥0.01	≥0.01

**Table 3:** Comparison of mandibular third molar extraction postoperative complications based on sex.

Gender (*N*)	Complication							
	Pain		Edema		Mouth opening restriction		Paresthesia	
	Day-1	Day-7	Day-1	Day-7	Day-1	Day-7	Day-1	Day-7
Female (540)	480 (88.9%)	6 (1.1%)	445 (82.4%)	8 (1.5%)	142 (26.3%)	4 (0.7%)	10 (1.9%)	4 (0.7%)
Male (376)	329 (87.5%)	5 (1.3%)	298 (79.3%)	7 (1.9%)	107 (28.5%)	4 (1.1%)	7 (1.9%)	1 (0.3%)
*P* values	>0.05	>0.05	>0.05	>0.05	>0.05	>0.05	>0.05	>0.05

**Table 4:** Comparison of mandibular third molar extraction postoperative complications based on the surgical difficulty level.

Difficulty (*N*)	Complication							
	Pain		Edema		Mouth opening restriction		Paresthesia	
	Day-1	Day-7	Day-1	Day-7	Day-1	Day-7	Day-1	Day-7
Simple (191)	149 (78%)	1 (0.5%)	153 (80.1%)	4 (2.1%)	41 (21.5%)	1 (0.5%)	3 (1.6%)	0 (0%)
Advanced (705)	644 (91.3%)	9 (1.3%)	573 (81.3%)	10 (1.4%)	197 (27.9%)	7 (1%)	10 (1.4%)	5 (0.7%)
Complex (20)	16 (80%)	1 (5%)	17 (85%)	1 (5%)	11 (55%)	0 (0%)	4 (20%)	0 (0%)
*P* values	≤0.001	≥0.01	≥0.01	≥0.01	≥0.01	≥0.01	≤0.001	≥0.01

## Data Availability

The research instruments were patients' medical records in addition to patients' panoramic photos needed to support the diagnosis. The research sample included patients with a history of impacted mandibular third molar surgical extraction using local anesthesia and split technique using a low-speed straight handpiece in the period between July 2017 and July 2019. The descriptive data were analyzed using the Chi-Square test with a *P* value of 0.05 with IBM Statistical Package for the Social Sciences (SPSS), Version 25.0 (IBM Corp., Armonk, New York, USA).
